# Supplementary material for: Spatiotemporal imaging of valence electron motion
Source: Nat Commun. 2019 Mar 5;10:1042. doi: 10.1038/s41467-019-09036-w (PMC6401056; doi:10.1038/s41467-019-09036-w)
Supplement: Supplementary file 1 — Supplementary Information [file 41467_2019_9036_MOESM1_ESM.pdf]

Supplementary Information
Spatiotemporal imaging of valence electron motion

M. Kübel, et al.*

Supplementary Note 1: Modulation in the Ar^{2+} yield

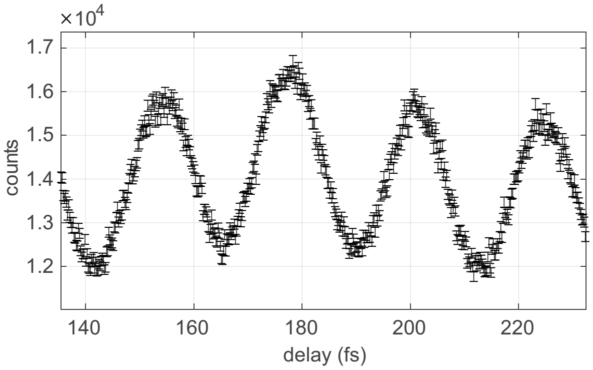

Supplementary Figure 1. Measured Ar^{2+} yield as a function of pump probe delay for several cycles of the spin-orbit period. Source data are provided as a Source Data file.

In Supplementary Figure 1, we present the recorded Ar^{2+} yield over the entire delay range studied in the experiment. The graph includes all instances where a Ar^{2+} ion was detected in coincidence with one electron. The modulation period is $T_{\rm SO} = (23.5 \pm 0.2)$ fs, in good agreement with the expected period of 23.3 fs [1]. The slightly higher Ar^{2+} yield, measured around $\Delta t = 180$ fs than elsewhere, is attributed to the small influence of the infrared deflection field on the ionization probability. Note that the delay between pump pulse and deflection field is kept constant, while the probe pulse is delayed with respect to the other two pulses. An active stabilization between probe and deflection pulses would allow for delaying the pump pulse instead, avoiding the influence of the deflection pulse envelope on the Ar^{2+} yield.

Supplementary Note 2: Calculated electron density snapshots

Momentum wave functions for the Ar^{+} valence orbital are obtained as described in Supplementary Method 2 below. The momentum space electron densities, $\Phi_m^2(p_x, p_y)$, in the $m = 0$ and $|m| = 1$ orbitals are given by the modulus square of the wave functions. The distortion caused by the deflection field is taken into account by convolution of P_m with a Gaussian, as given by equation 7.

The time-dependent population in the $m = 0$ states is given by [2]

$$\begin{aligned} P_0(\Delta t) &= 13/9 - H(\Delta t), \\ P_1(\Delta t) &= 32/9 + H(\Delta t), \end{aligned} \tag{1}$$

where H is the time-dependent hole density

$$H(\Delta t) = 4/9 \cos\left(2\pi \Delta t / T_{\rm SO}\right) \tag{2}$$

The momentum space snapshots are obtained by calculating the normalized difference between the time-dependent electron distribution,

$$S = P_0(\Delta t)\Phi_0^2 + P_1(\Delta t)\Phi_1^2, \tag{3}$$

and the time-averaged one,

$$R = 13/9\Phi_0^2 + 32/9\Phi_1^2. \tag{4}$$

In Supplementary Figure 2, we present calculated electron density plots at several fractions of the spin orbit period, including 4/6 and $5/6\,T_{\rm SO}$ that are not shown in Fig. 2 of the main text. They differ from the ones at 1/2 and $1\,T_{\rm SO}$, respectively, by their contrast but are otherwise qualitatively identical. This is consistent with the evolution of the wave packet described by equations 1 [2].

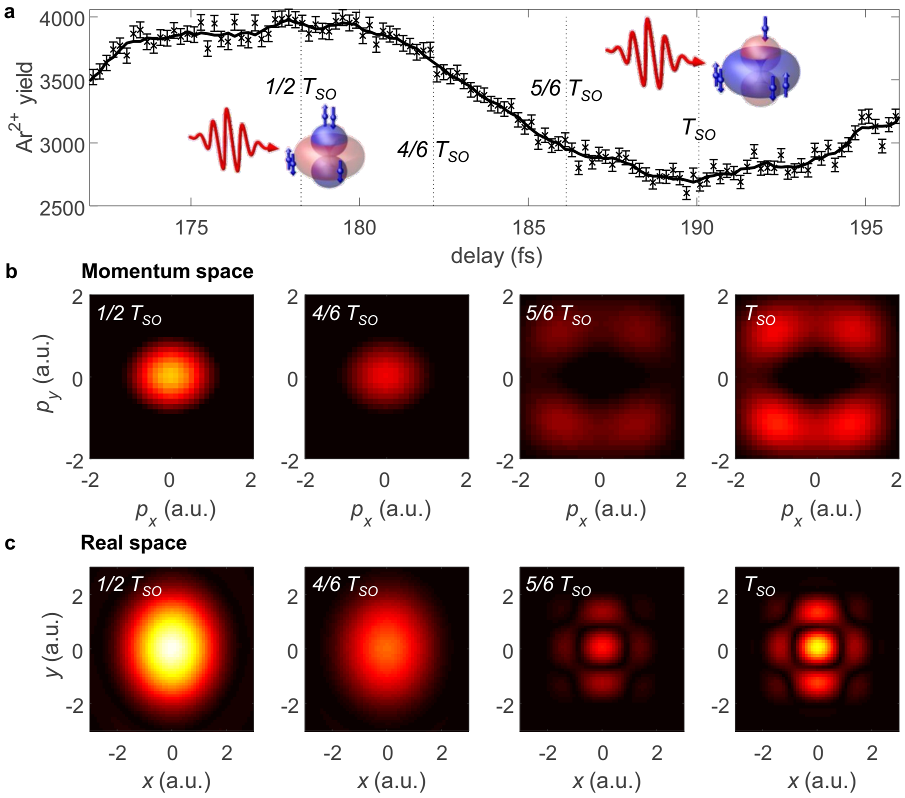

Supplementary Figure 2. Calculated snapshots of the spin-orbit wave packet in Ar^+. Similar to Fig. 2 of the main text but showing only calculated data, including intermediate delays not shown in Fig. 2. Source data are provided as a Source Data file.

Supplmentary Method 1: Selecting the photoelectrons from Ar^+ ionization

Supplementary Figure 3 shows the two-electron momentum spectrum for the two electrons emitted from Ar^{2+}. Here, the ion yield is plotted as a function of the detected and calculated momentum components along the polarization of the mid-IR deflection field. The two-electron spectrum is a graphical way to identify the events for which the first ionization has taken place in the pump step, and the probe pulse removes an electron from Ar^+. These are marked by the green oval. In this case, the measured electron has a large negative momentum along the IR polarization, which can only originate from the probe pulse. The calculated momentum component of the electron, which was not detected, is small, and was therefore most likely produced by the pump pulse.

We only choose events where the detected electron is deflected towards the electron detector (i.e. negative momenta), because electrons deflected away from the detector (i.e. positive momenta) are more often subject to false coincidences. Events originating from either of the pulses alone are also marked in the plot. We note that the events highlighted by the black oval permit isolating the electron removed from neutral Ar in the pump pulse. These events would permit orbital imaging of a molecular target that cannot be pre-aligned.

Supplmentary Method 2: Imaging model

The theoretical images shown in Fig. 4(b) are obtained by the following procedure. Real space wave functions for the Ar^+ valence orbital are taken from the computational chemistry software GAMESS. Momentum-real space wave functions are calculated by partial Fourier transform, as described in Ref. [3], and plotted in Fig. 4(c). The projections of these wave functions to the $z = 0$ plane are used for the simple calculations presented in Fig. 2 of the main text.

The wavefunctions squared are multiplied with a “tunnel filter” [3] to obtain the transversal momentum distribution at the tunnel exit,

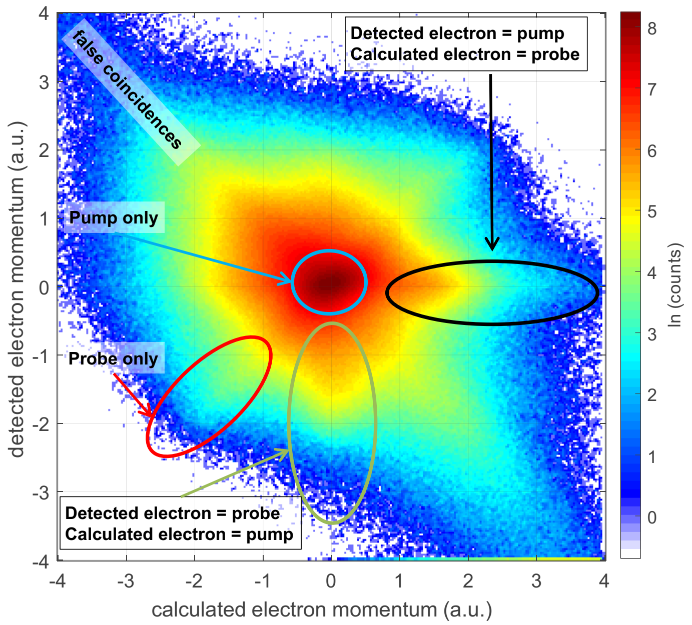

Supplementary Figure 3. Identification of ionization pathways leading to Ar^+. The two-electron spectrum show the Ar^{2+} yield as a function of both electron momenta along the mid-IR polarization. It allows for distinguishing different types of coincidence events. The events in the green oval permit us to image the spin-orbit wave packet in Ar^+, and were used to generate Figs. 2, 3 and 4. Source data are provided as a Source Data file.

$$\Psi_m(z_e, p_x, p_y)^2 = \Psi_m(z_0, p_x, p_y)^2 \times f, \tag{5}$$

where we use the tunnel entrance for z_0. The filter function is given by

$$f = \exp(-0.5(p_x^2 + p_y^2) \times \tau), \tag{6}$$

where $\tau = \sqrt{I_P/(2U_P)}/\omega$, with the ionization potential I_P, the ponderomotive potential U_P, and the laser frequency ω. The longitudinal momentum at the tunnel exit z_e is is assumed to equal zero.

In order to obtain the momentum distributions Φ_m^2 after propagation in the laser field, the momentum distributions at the tunnel exit are convoluted with Gaussian functions,

$$\Phi_m^2 = \frac{1}{2\pi w_x w_z} \left(\Psi_\mathrm{m}(z_e, p_x, p_y)^2 * \exp\left(-\frac{(p_x - p_0)^2}{w_x^2} \right) \right) * \exp\left(-\frac{p_z^2}{w_z^2} \right), \tag{7}$$

where the values for $p_0 = 1.6\,\text{a.u.}$ (deflection amplitude), $w_x = 0.35\,\text{a.u.}$, and w_z are obtained from simultaneously measured photoelectron momentum spectra recorded for single ionization of Ar^+. For the $m = 0$ orbital, we use $w_z^p = 1.23\,\text{a.u.}$, for the $|m| = 1$ orbital, we use $w_z^d = 1.20\,\text{a.u.}$. The ratio w_z^p/w_z^d was obtained from the ionization rate calculations described in the Methods section *Orbital effect in the longitudinal momentum spectra*.

The momentum distribution that correspond to $m = 0$ and $|m| = 1$ *vacancies* are calculated by

$$S_0 = \Phi_0^2 + 4\Phi_1^2 \tag{8}$$
$$S_1 = 2\Phi_0^2 + 3\Phi_1^2. \tag{9}$$

To calculate the normalized differences in the three momentum planes, S_0 and S_1 are integrated over the third dimension.

Supplementary References

* matthias.kuebel@uni-jena.de

[1] Fleischer, A. *et al.* Probing Angular Correlations in Sequential Double Ionization. *Phys. Rev. Lett.* **107**, 113003 (2011).
[2] Wörner, H. J. & Corkum, P. B. Imaging and controlling multielectron dynamics by laser-induced tunnel ionization. *J. Phys. B At. Mol. Opt. Phys.* **44**, 041001 (2011).
[3] Murray, R., Spanner, M., Patchkovskii, S. & Ivanov, M. Y. Tunnel ionization of molecules and orbital imaging. *Phys. Rev. Lett.* **106**, 173001 (2011).
