# Supplementary material for: Spatiotemporal imaging of valence electron motion
Source: Nat Commun. 2019 Mar 5;10:1042. doi: 10.1038/s41467-019-09036-w (PMC6401056; doi:10.1038/s41467-019-09036-w)
Supplement: Supplementary file 3 — Description of Additional Supplementary Files [file 41467_2019_9036_MOESM3_ESM.docx]

**Description of Additional Supplementary Files**

**File Name: Supplementary Movie 1**

**Description:** “Electron_wavepacket_2D_movie.avi”. Two-dimensional movie of a spin-orbit wave packet in the argon cation. (Top) The measured time-dependent yield of Ar2+ is modulated with the spin-orbit period. For the part of the yield curve highlighted in red, the recorded photoelectron momentum distribution (PMD) is compared to the delay-averaged PMD to obtain the electron density plot below. (bottom) Measured variation in the valence electron density. The revivals of the ring (spot) shape at the yield minima (maxima) indicates localization of the electron hole in the m=0 (|m|=1) states. The expected circular symmetry is broken by a noticeable stretch along the x axis, induced by the streaking laser pulse.

**File Name: Supplementary Movie 2**

**Description:** “Electron_wavepacket_3D_movie.avi”. Three-dimensional movie of a spin-orbit wave packet in the argon cation. The top panel shows the measured time-dependent yield of Ar2+, modulated with the spin-orbit period. For the part of the yield curve highlighted in red, the recorded photoelectron momentum distribution (PMD) is compared to the delay-averaged PMD to obtain the electron density plot below. The bottom panel shows the normalized differences in the projections of the measured three-dimensional photoelectron momentum distribution. In each plane the data is integrated over the perpendicular dimension.
